# Supplementary material for: Comparative Analysis of the Genetic Composition of Minorities in the Carpathian Basin Through Genome-Wide Autosomal Data
Source: Genes (Basel). 2025 May 21;16(5):607. doi: 10.3390/genes16050607 (PMC12111567; doi:10.3390/genes16050607)

Tracy-Widom statistics of the PCA of dataset 1 (with 1KGP Europe reference).					
**eigenvector**	**eigenvalue**	**difference**	**twstat**	**p-value**	**effective n**
1	3.52836	NA	299.2	0	7888.378
2	2.766758	-0.761602	164.653	0	8132.845
3	1.9906	-0.776158	18.569	4.62E-25	8244.768
4	1.755973	-0.234627	-26.167	1	8265.341

Variance of the PCA of dataset 1 (with 1KGP Europe reference).		
**PC**	**ExplainedVariance(%)**	**CumulativeVariance(%)**
PC1	42.5836	42.5836
PC2	33.3919	75.9755
PC3	24.0245	100

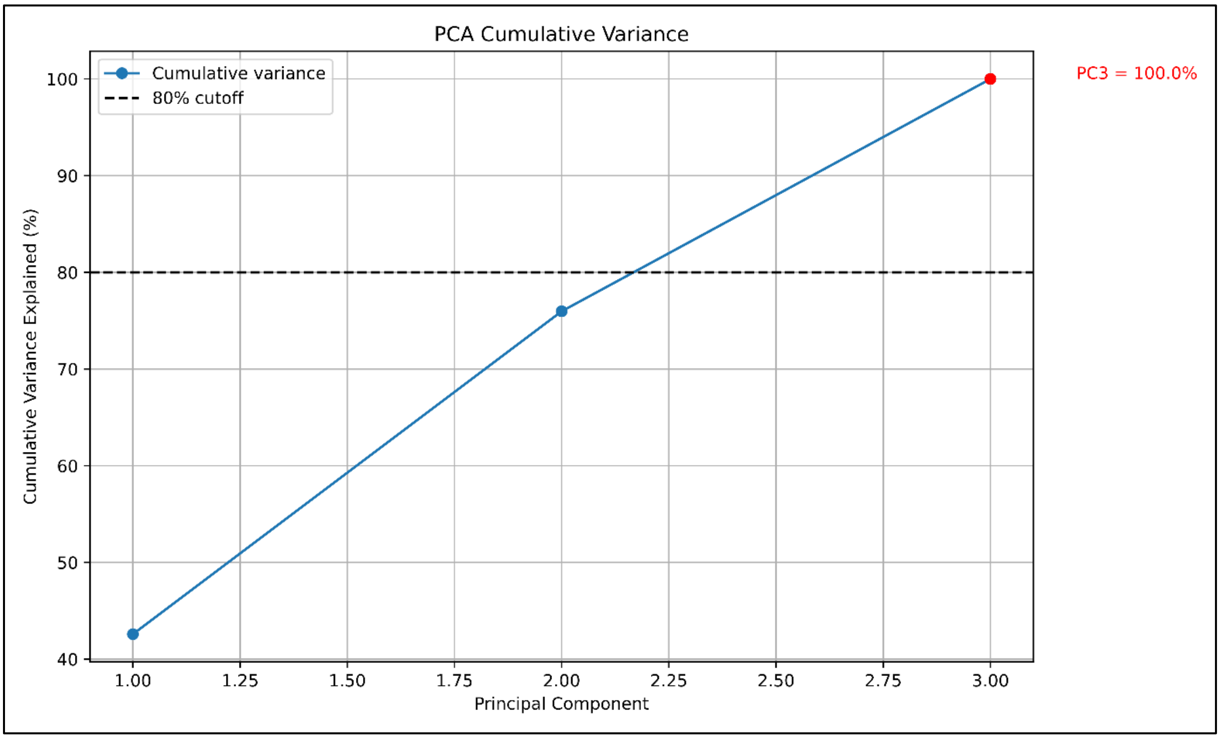

Tracy-Widom statistics of the PCA of dataset 2 (with HGDP Europe, and HGDP Central, and South Asia references).					
**eigenvector**	**eigenvalue**	**difference**	**twstat**	**p-value**	**effective n**
1	11.052738	NA	1578.699	0	6990.7
2	3.298911	-7.753827	718.804	0	23927.607
3	2.319896	-0.979015	386.162	0	27378.725
4	1.844226	-0.47567	197.715	0	28701.282
5	1.761965	-0.08226	166.51	0	29239.293
6	1.6462	-0.115766	119.022	0	29683.397
7	1.574573	-0.071627	89.478	4.01E-247	29993.843
8	1.559597	-0.014975	84.467	8.01E-227	30231.294
9	1.545889	-0.013708	79.899	7.84E-209	30458.65
10	1.540023	-0.005866	78.761	1.98E-204	30676.953
11	1.532989	-0.007035	77.061	6.59E-198	30894.305
12	1.523346	-0.009643	74.127	7.94E-187	31109.331
13	1.517721	-0.005625	72.965	1.71E-182	31319.055
14	1.504087	-0.013634	68.088	1.06E-164	31527.626
15	1.496183	-0.007904	65.752	2.13E-156	31726.233
16	1.487284	-0.008899	62.903	1.80E-146	31920.81
17	1.479501	-0.007783	60.514	2.57E-138	32110.125
18	1.462751	-0.01675	53.884	1.57E-116	32295.209
19	1.461133	-0.001618	54.23	1.23E-117	32466.366
20	1.442587	-0.018546	46.607	4.05E-94	32639.408
21	1.439606	-0.002981	46.207	6.21E-93	32796.443
22	1.417884	-0.021722	36.915	6.62E-67	32953.526
23	1.408385	-0.0095	33.287	1.46E-57	33091.338
24	1.391455	-0.01693	26.064	1.86E-40	33222.221
25	1.386287	-0.005168	24.363	9.70E-37	33338.868
26	1.370004	-0.016283	17.313	9.60E-23	33452.62
27	1.365307	-0.004697	15.739	5.92E-20	33552.911
28	1.360078	-0.00523	13.889	7.55E-17	33650.56
29	1.354986	-0.005092	12.083	5.23E-14	33745.034
30	1.350535	-0.004451	10.565	8.92E-12	33836.416
31	1.345762	-0.004773	8.875	1.81E-09	33925.257
32	1.340812	-0.004951	7.079	1.10E-07	34011.226
33	1.337198	-0.003613	5.913	2.42E-06	34094.127
34	1.33195	-0.005249	3.939	0.000251425	34175.138
35	1.328064	-0.003886	2.608	0.00357216	34252.753
36	1.32539	-0.002674	1.855	0.0132387	34328.168
37	1.324918	-0.000472	2.17	0.00779165	34402.442
38	1.32013	-0.004788	0.374	0.109658	34477.542

39	1.318403	-0.001727	0.062	0.157161	34549.571
40	1.318077	-0.000326	0.434	0.102012	34621.255
41	1.313607	-0.00447	-1.23	0.487624	34693.853
42	1.309551	-0.004056	-2.705	0.88731	34763.61
43	1.307941	-0.001611	-2.989	0.929011	34830.873
44	1.306314	-0.001627	-3.283	0.959126	34897.82
45	1.305341	-0.000973	-3.258	0.956976	34964.426
46	1.303139	-0.002202	-3.841	0.9885	35031.277
47	1.299938	-0.003201	-4.927	0.999631	35097.253
48	1.299158	-0.000779	-4.819	0.999446	35161.428
49	1.294759	-0.0044	-6.512	1	35225.986

Variance of the PCA of dataset 2 (with HGDP Europe, and HGDP Central, and South Asia references).		
**PC**	**ExplainedVariance(%)**	**CumulativeVariance(%)**
PC1	16.6884	16.6884
PC2	4.981	21.6693
PC3	3.5028	25.1721
PC4	2.7846	27.9567
PC5	2.6604	30.617
PC6	2.4856	33.1026
PC7	2.3774	35.48
PC8	2.3548	37.8349
PC9	2.3341	40.169
PC10	2.3253	42.4942
PC11	2.3146	44.8089
PC12	2.3001	47.1089
PC13	2.2916	49.4005
PC14	2.271	51.6715
PC15	2.2591	53.9306
PC16	2.2456	56.1762
PC17	2.2339	58.4101
PC18	2.2086	60.6187
PC19	2.2061	62.8248
PC20	2.1781	65.003
PC21	2.1736	67.1766
PC22	2.1408	69.3174
PC23	2.1265	71.4439
PC24	2.1009	73.5449
PC25	2.0931	75.638

PC26	2.0685	77.7066
PC27	2.0615	79.768
PC28	2.0536	81.8216
PC29	2.0459	83.8674
PC30	2.0392	85.9066
PC31	2.0319	87.9385
PC32	2.0245	89.963
PC33	2.019	91.982
PC34	2.0111	93.9931
PC35	2.0052	95.9983
PC36	2.0012	97.9995
PC37	2.0005	100

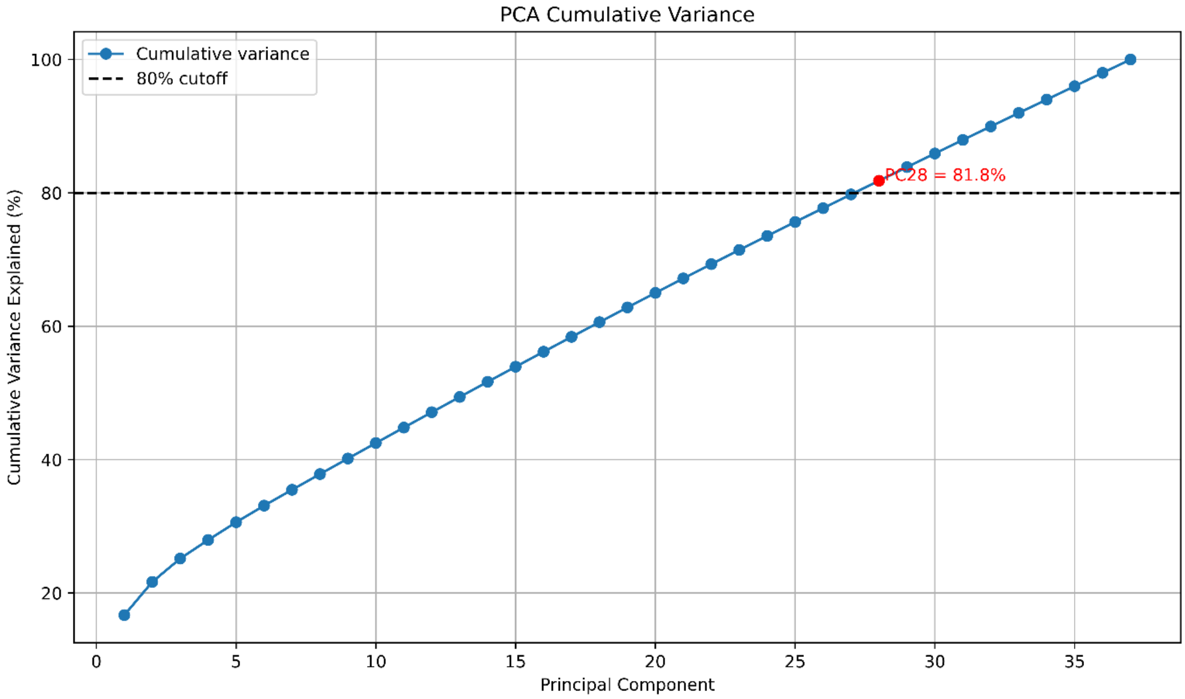

Supplement: Supplementary file 1 [file genes-16-00607-s001.zip › Table_S1.pdf]
