# Supplementary material for: Comparative Analysis of the Genetic Composition of Minorities in the Carpathian Basin Through Genome-Wide Autosomal Data
Source: Genes (Basel). 2025 May 21;16(5):607. doi: 10.3390/genes16050607 (PMC12111567; doi:10.3390/genes16050607)
Supplement: Supplementary file 1 [file genes-16-00607-s001.zip › Table_S2.pdf]

W<br>Population 1	X<br>Population 2	Y<br>Population 3	Z<br>Population 4	D-statistics	Z-score
Yoruba	Hungarian	Romanian	ArpadHun	0.000303	**2.316**
Yoruba	Hungarian	Romanian	TransHun	0.000139	1.17
Yoruba	Hungarian	Romanian	KorondSekler	-0.000105	-0.84
Yoruba	Hungarian	Romanian	TransSekler	0.000012	0.104
Yoruba	Hungarian	Romanian	BukovinaSekler	0.000262	**2.122**
Yoruba	Hungarian	Romanian	GyergyoSekler	-0.000024	-0.227
Yoruba	Hungarian	Romanian	HavadSekler	0.000266	**2.165**
Yoruba	Hungarian	Romanian	KezdivasSekler	0.000066	0.568
Yoruba	Hungarian	Romanian	NyaradmenteSekler	0.000143	1.165
Yoruba	Hungarian	Romanian	SzekelykocsSekler	0.000044	0.318
Yoruba	Hungarian	Romanian	SzekelyudvSekler	0.000044	0.349
Yoruba	Hungarian	Romanian	MoldavianCsango	0.000131	1.054
Yoruba	Hungarian	Romanian	GyimesCsango	0.000091	0.788
Yoruba	Hungarian	German	Swabian	0.000073	0.609
Yoruba	MoldavianCsango	Romanian	ArpadHun	0.000323	**2.445**
Yoruba	MoldavianCsango	Romanian	TransHun	0.000096	0.802
Yoruba	MoldavianCsango	Romanian	KorondSekler	-0.000056	-0.449
Yoruba	MoldavianCsango	Romanian	TransSekler	0.000012	0.106
Yoruba	MoldavianCsango	Romanian	BukovinaSekler	0.000242	1.957
Yoruba	MoldavianCsango	Romanian	GyergyoSekler	0.00003	0.284
Yoruba	MoldavianCsango	Romanian	HavadSekler	0.00022	1.792
Yoruba	MoldavianCsango	Romanian	KezdivasSekler	0.000096	0.813
Yoruba	MoldavianCsango	Romanian	NyaradmenteSekler	0.000167	1.343
Yoruba	MoldavianCsango	Romanian	SzekelykocsSekler	0.00003	0.219
Yoruba	MoldavianCsango	Romanian	SzekelyudvSekler	0.000054	0.428
Yoruba	MoldavianCsango	Romanian	GyimesCsango	0.000278	**2.405**
Yoruba	GyimesCsango	Romanian	ArpadHun	0.000143	1.067
Yoruba	GyimesCsango	Romanian	TransHun	-0.000012	-0.101
Yoruba	GyimesCsango	Romanian	KorondSekler	-0.000233	-1.845
Yoruba	GyimesCsango	Romanian	TransSekler	-0.000167	-1.476
Yoruba	GyimesCsango	Romanian	BukovinaSekler	0.00011	0.891
Yoruba	GyimesCsango	Romanian	GyergyoSekler	-0.000093	-0.862
Yoruba	GyimesCsango	Romanian	HavadSekler	0.00008	0.638
Yoruba	GyimesCsango	Romanian	KezdivasSekler	-0.000073	-0.623
Yoruba	GyimesCsango	Romanian	NyaradmenteSekler	0.000014	0.113
Yoruba	GyimesCsango	Romanian	SzekelykocsSekler	-0.000081	-0.584
Yoruba	GyimesCsango	Romanian	SzekelyudvSekler	-0.000072	-0.575
Yoruba	GyimesCsango	Romanian	MoldavianCsango	0.000173	1.388
