# Supplementary figures and images for: Comparative Analysis of the Genetic Composition of Minorities in the Carpathian Basin Through Genome-Wide Autosomal Data
Source: Genes (Basel). 2025 May 21;16(5):607. doi: 10.3390/genes16050607 (PMC12111567; doi:10.3390/genes16050607)

K=10

K=9

K=8

K=7

K=6

K=5

K=4

K=3

K=2

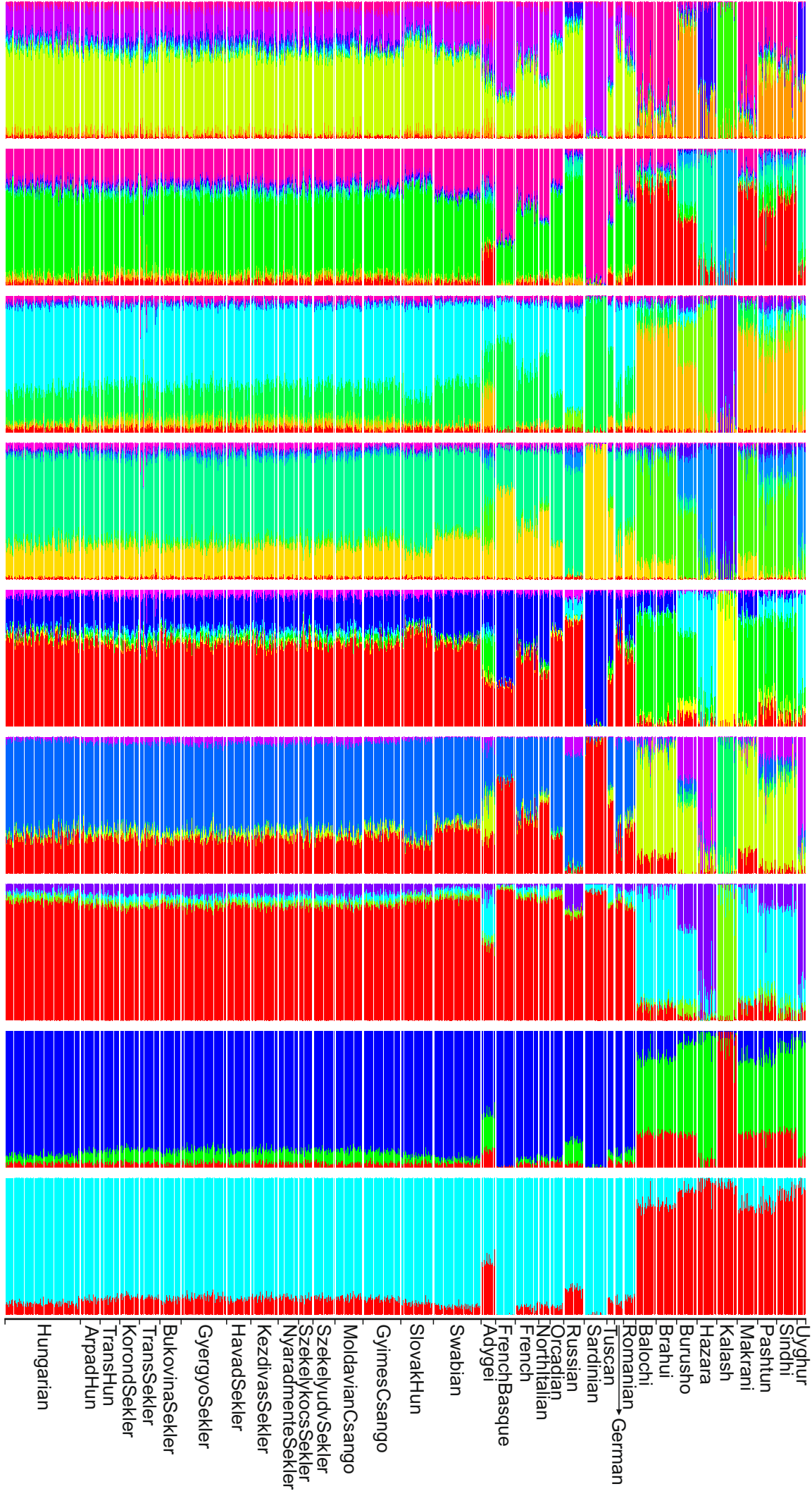

Hungarian speaking ethnicities

Supplement: Supplementary file 1 [file genes-16-00607-s001.zip › Figure_S1.pdf]

K=10

K=9

K=8

K=7

K=6

K=5

K=4

K=3

K=2

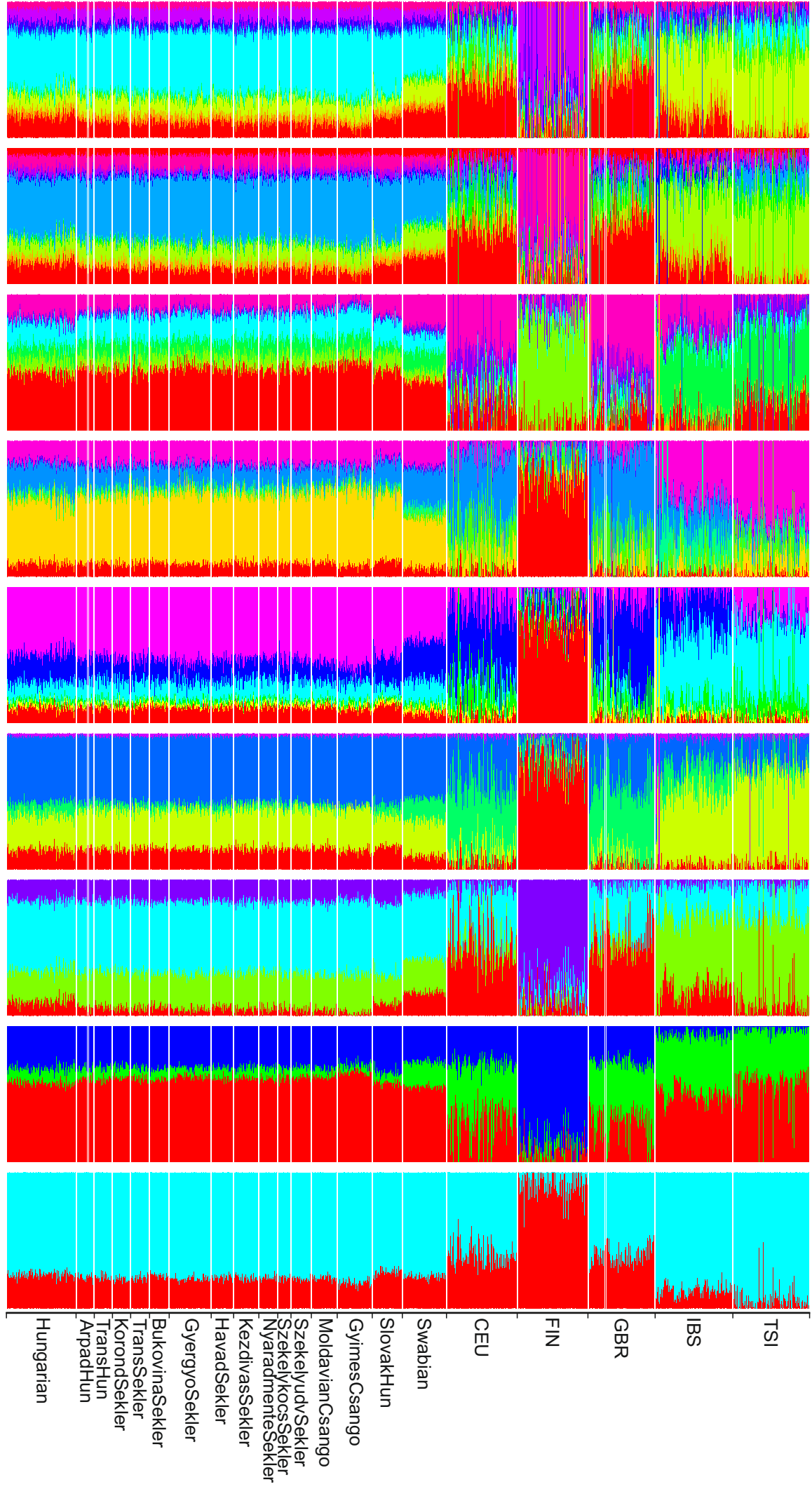

Hungarian speaking ethnicities

Supplement: Supplementary file 1 [file genes-16-00607-s001.zip › Figure_S2.pdf]

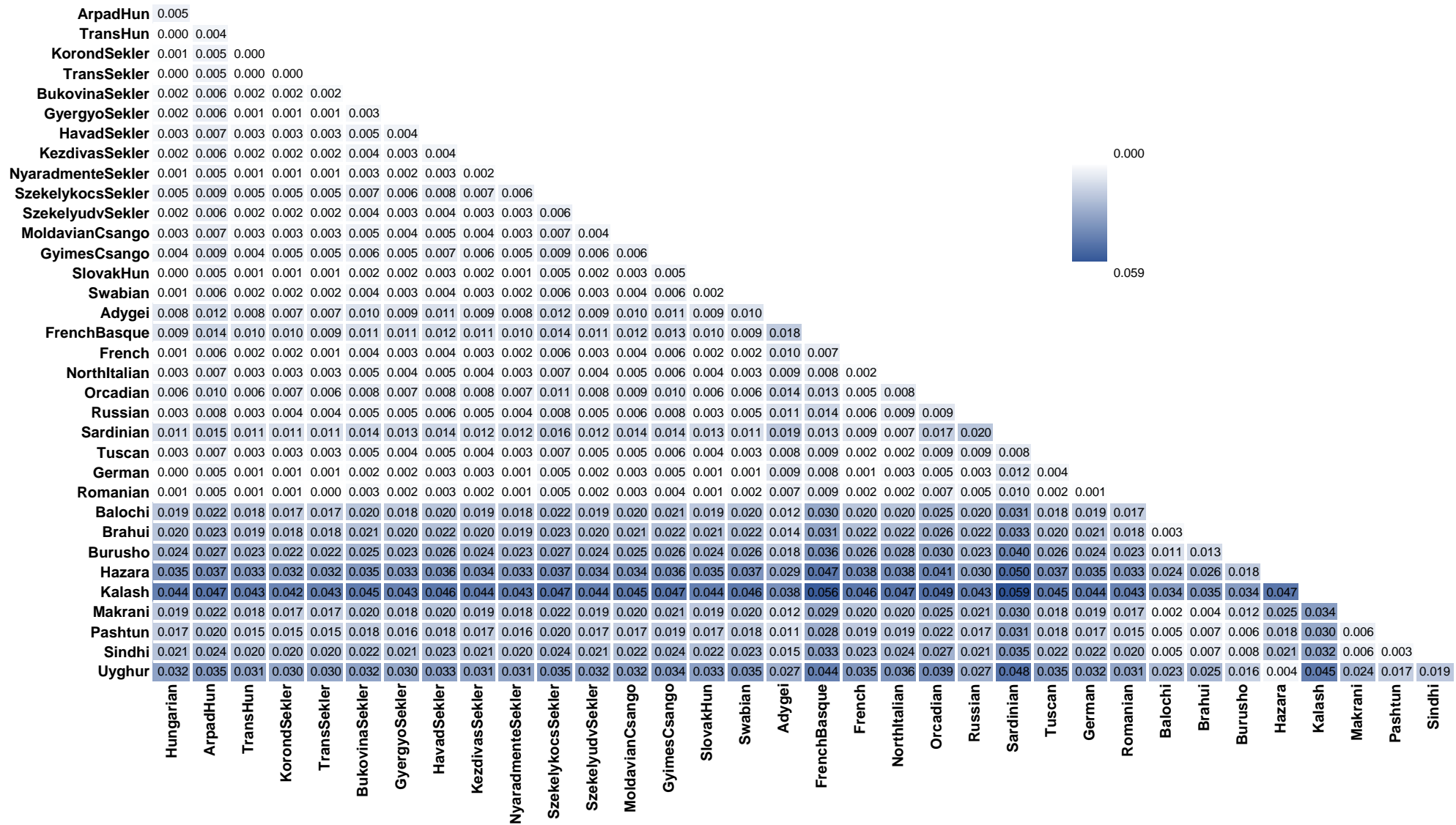

Supplement: Supplementary file 1 [file genes-16-00607-s001.zip › Figure_S3.pdf]

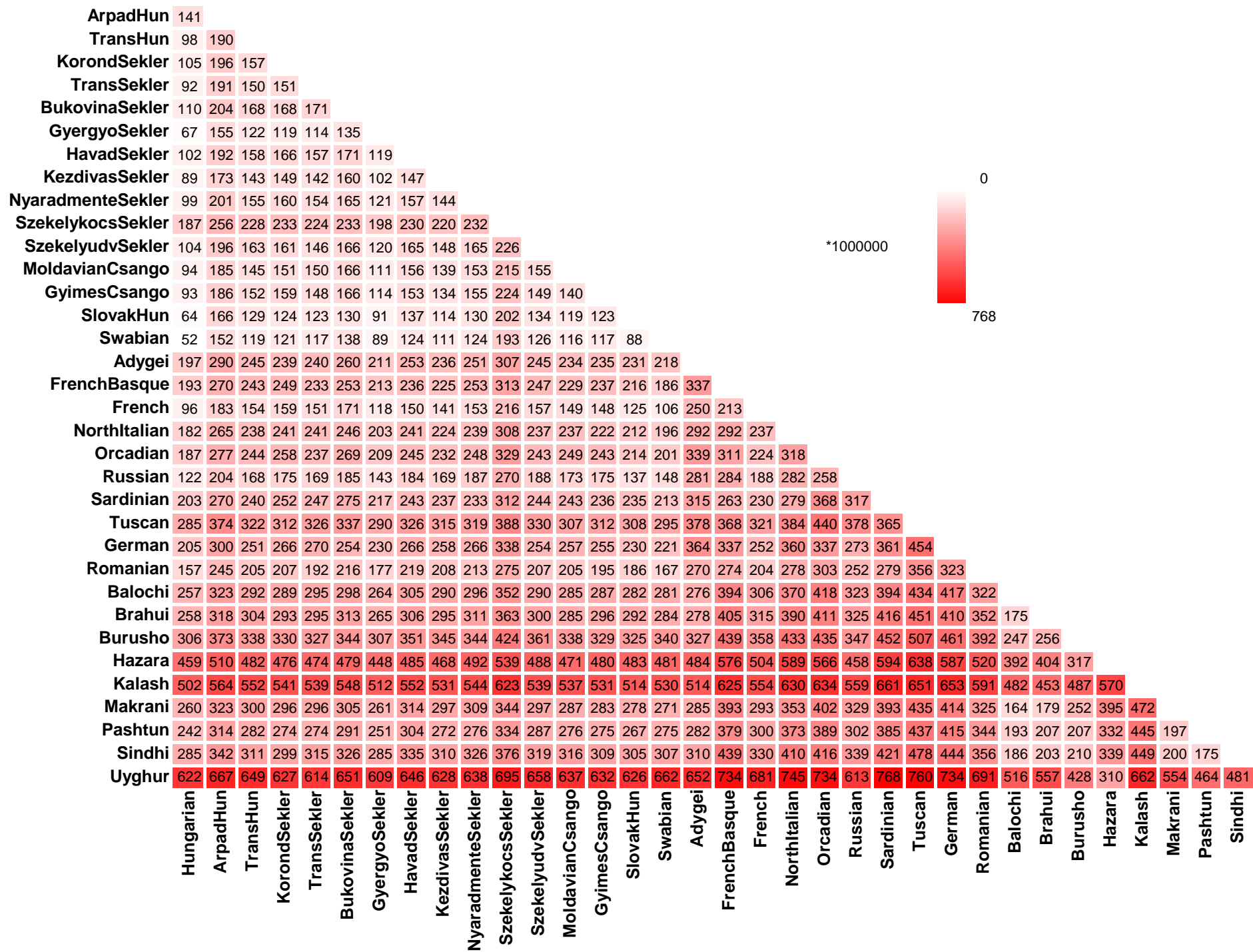

Supplement: Supplementary file 1 [file genes-16-00607-s001.zip › Figure_S4.pdf]

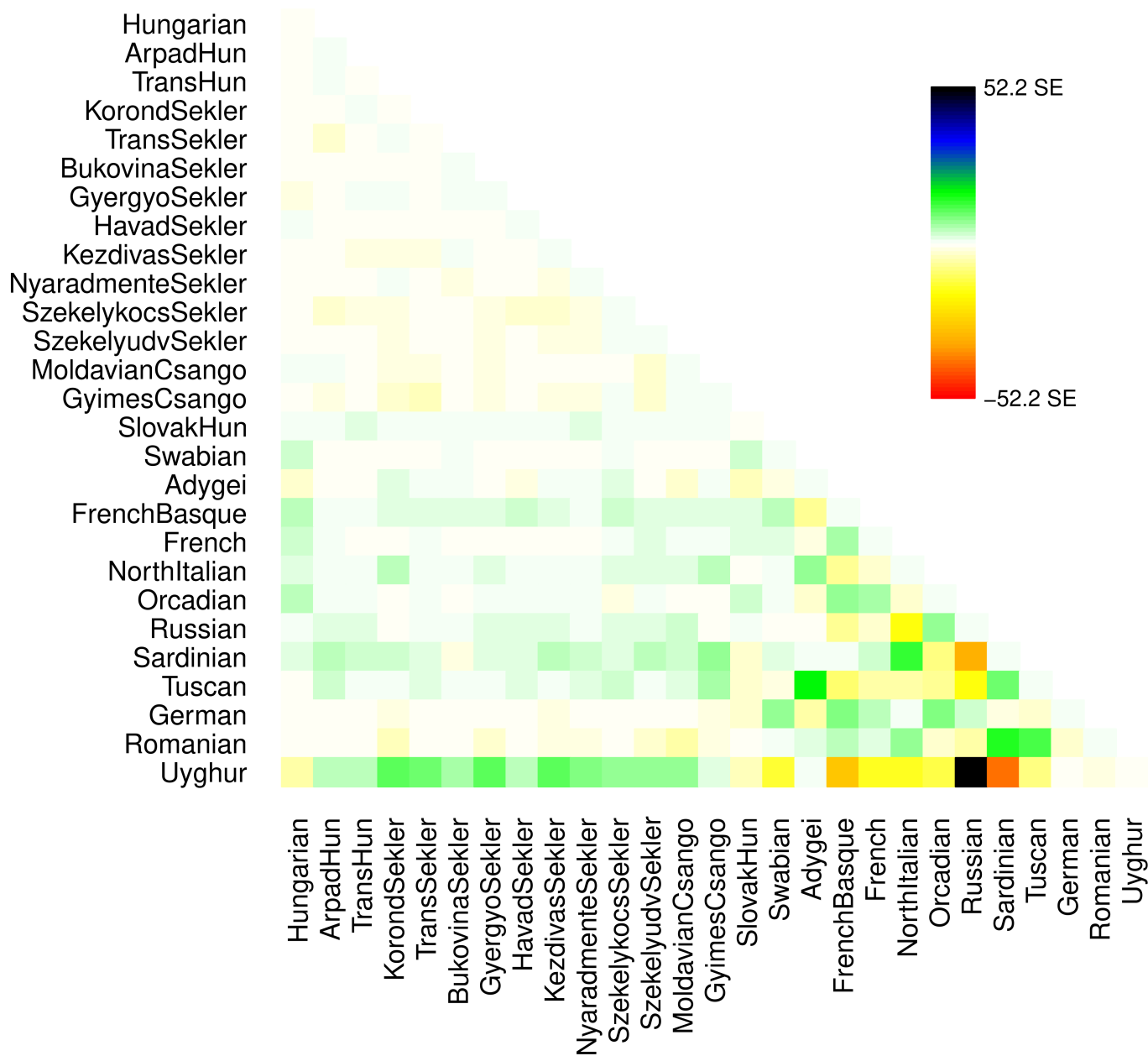

Supplement: Supplementary file 1 [file genes-16-00607-s001.zip › Figure_S5.pdf]
